# The Replacement of Only One Portion of Starchy Carbohydrates with Green Leafy Vegetables Regresses Mid and Advanced Stages of NAFLD: Results from a Prospective Pilot Study

**DOI:** 10.3390/nu15102289

**Published:** 2023-05-12

**Authors:** Sara De Nucci, Roberta Rinaldi, Martina Di Chito, Rossella Donghia, Vito Giannuzzi, Endrit Shahini, Raffaele Cozzolongo, Pasqua Letizia Pesole, Sergio Coletta, Giovanni De Pergola, Gianluigi Giannelli

**Affiliations:** 1 Unit of Geriatrics and Internal Medicine, National Institute of Gastroenterology—IRCCS “Saverio de Bellis”, Via Turi 27, 70013 Castellana Grotte, Bari, Italy; 2 Unit of Data Science, National Institute of Gastroenterology—IRCCS “Saverio de Bellis”, Via Turi 27, 70013 Castellana Grotte, Bari, Italy; 3 Department of Gastroenterology, National Institute of Gastroenterology—IRCCS “Saverio de Bellis”, Via Turi 27, 70013 Castellana Grotte, Bari, Italy; 4 National Institute of Gastroenterology—IRCCS “Saverio de Bellis”, Via Turi 27, 70013 Castellana Grotte, Bari, Italy; 5 Scientific Direction, National Institute of Gastroenterology—IRCCS “Saverio de Bellis”, Via Turi 27, 70013 Castellana Grotte, Bari, Italy

**Keywords:** green leafy vegetables, non-alcoholic fatty liver disease (NAFLD), transient elastography, FibroScan

## Abstract

The gold standard treatment for NAFLD is weight loss and lifestyle interventions, which require a diet enriched in fiber and reduced in sugars and saturated fats. Fibres may be advantageous for NAFLD patients since they reduce and slow the absorption of carbohydrates, lipids, and proteins, lowering the energy density of the meal and increasing their sense of satiety. Furthermore, the polyphenol content and other bioactive compounds of vegetables have antioxidant and anti-inflammatory properties preventing disease progression. The aim of this study is to ascertain the effects of a diet enriched by green leafy vegetables and with a moderate restriction of carbohydrate intake in patients with NAFLD over a three month period. Among the forty patients screened, twenty four patients completed the clinical trial consisting of swapping one portion of carbohydrate-rich food for one portion of green leafy vegetables, and liver and metabolic markers of NAFLD were evaluated. All patients underwent routine blood tests, anthropometric measurements, bioelectrical impedance analysis, fibroscan, and fatty liver index (FLI) evaluation before and at the end of the study. The population under study (*n* = 24) had a median age of 47.5 (41.5–52.5) years and included mainly women (70.8%). We found that FLI, which is used to predict fatty liver (73 (33–89) vs. 85 (54–95), *p* < 0.0001) and the FAST score, which is a fibroscan-derived parameter identifying patients at risk of progressive NASH (0.03 (0.02–0.09) vs. 0.05 (0.02–0.15), *p* = 0.007), were both improved after changes in diet. The BMI (33.3 (28.6–37.3) vs. 35.3 (31.2–39.0), *p* < 0.0001), WC (106.5 (95.0–112.5) vs. 110.0 (103.0–124.0), *p* < 0.0001), neck circumference (38.0 (35.0–41.5) vs. 39.5 (38.0–42.5), *p* < 0.0001), fat mass (32.3 (23.4–40.7) vs. 37.9 (27.7–43.5), *p* < 0.0001), and extracellular water (17.3 (15.2–20.8) vs. 18.3 (15.9–22.7), *p* = 0.03) were also all significantly lower after three months of diet. Metabolic parameters linked to NAFLD decreased: HbA1c (36.0 (33.5–39.0) vs. 38.0 (34.0–40.5), *p* = 0.01), triglycerides (72 (62–90) vs. 90 (64–132), *p* = 0.03), and the liver markers AST (17 (14–19) vs. 18 (15–27), *p* = 0.01) and γGT (16 (13–20) vs. 16 (14–27), *p* = 0.02). In conclusion, replacing only one portion of starchy carbohydrates with one portion of vegetables for a three month period is sufficient to regress, at least in part, both mid and advanced stages of NAFLD. This moderate adjustment of lifestyle habits is easily achievable.

## 1. Introduction

Non-alcoholic fatty liver disease (NAFLD) is the leading cause of liver disease globally, and its prevalence rose in Italy from 25% in 2016 to 30% in 2019, putting a considerable burden on the national healthcare system [1,2]. It encompasses a wide range of histological aspects, ranging from non-alcoholic fatty liver (NAFL) to non-alcoholic steatohepatitis (NASH), liver fibrosis, cirrhosis, and ultimately hepatocellular carcinoma. This progression has emerged as the most common cause of chronic liver damage, and one of the foremost indications for liver transplantation [3]. Furthermore, the prevalence is higher in individuals who suffer from metabolic disorders, and NAFLD is reported in 54–90% of subjects with obesity, especially the abdominal morphotype [4]. NAFLD is characterised by the presence of steatosis in more than 5% of hepatocytes in combination with metabolic risk factors (in particular, obesity and type 2 diabetes). The exclusion of excessive alcohol consumption (<30 g per day for men and 20 g per day for women) and of secondary causes is necessary for diagnosis. NAFLD is the liver expression of the metabolic syndrome related to obesity, and a high body mass index is a major risk factor for steatosis [5]. The recent concept of Metabolic dysfunction Associated with Fatty Liver Disease (MAFLD) emphasises the role of cardiometabolic risk factors in the onset and progression of liver disease [6]. Patients with NAFLD, particularly NASH, have higher liver-specific mortality rates but also increased overall mortality and risk of type 2 diabetes and chronic kidney disease [7]. 

The European Association for the Study of the Liver (EASL) suggests the benefit of a moderate reduction in carbohydrates and fat, following a Mediterranean diet pattern [8]. The Mediterranean diet is recognised as the most healthy diet for a population [9], being characterized by the intake of at least five portions of fruit and vegetables, with a rich fibre content, per day [10]. Due to their lower energy density and capacity to promote satiety, fibres may be advantageous for NAFLD patients, as they reduce and slow the absorption of carbohydrates and lipids. Fibers also have an impact on protein absorption and digestion, thus decreasing correlations of aminoacids with insulin resistance [11]. Moreover, fibres have prebiotic effects modifying gut microbial composition, which may contribute to inhibiting the development of NAFLD [12]. Fibres may also be involved in lowering the risk for NAFLD by increasing the production of short-chain fatty acids and phenolic compounds, which have antioxidant effects in the liver [13]. Additionally, dietary fibres decrease the risk of Metabolic Syndrome (MetS) components, such as type 2 diabetes and dyslipidemia, as well as cardiovascular risk. In general, the prevention and treatment of chronic diseases can benefit from the consumption of leafy vegetables as they are particularly rich in dietary fibre and antioxidants such as carotenoids, isothiocyanates, polyphenols, and vitamins [14]. In primary care, weight-reduction and lifestyle interventions, mainly involving diet and physical exercise, are the gold standard treatment for NAFLD [15]. However, there is still no dietary pattern globally recognised as the best suited one [16]. 

The aim of this study is to investigate the impact of a dietary intervention, consisting of replacing a daily serving of carbohydrates with a serving of vegetables, on NAFLD patients in a longitudinal prospective study.

## 2. Materials and Methods

### 2.1. Study Design and Population

The Clinical Nutrition Centre for Research and Treatment of Obesity and Metabolic Diseases of the National Institute of Gastroenterology Saverio de Bellis Research Hospital (Castellana Grotte, Bari, Italy) performed this three-month prospective study. The inclusion criteria were an age between 18 and 65 years, a BMI over 25, and not taking any medication. Subjects with any disease that could influence the presence of steatosis, such as IBS or IBD, pregnancy or lactation, and alcohol or substance abuse, were excluded. Overweight and obese patients recruited from our outpatient clinic underwent anthropometric and bioimpedance measurements, clinical history, and laboratory tests (haematology, biochemistry). To determine daily alcohol consumption, the explicit question was asked: “Do you drink more than two glasses of alcohol per day?” in males and “Do you drink more than one glass of alcohol per day?” in females, following the American and European guidelines on daily alcohol consumption of a threshold of 20 g/day for females and 30 g/day for males [17]. The International Physical Activity Questionnaire (IPAQ) was used to measure physical activity [18], and the PREDIMED questionnaire to quantify adherence to MD [19]. The subjects were questioned about a smoking habit, too.

**Figure 1:**
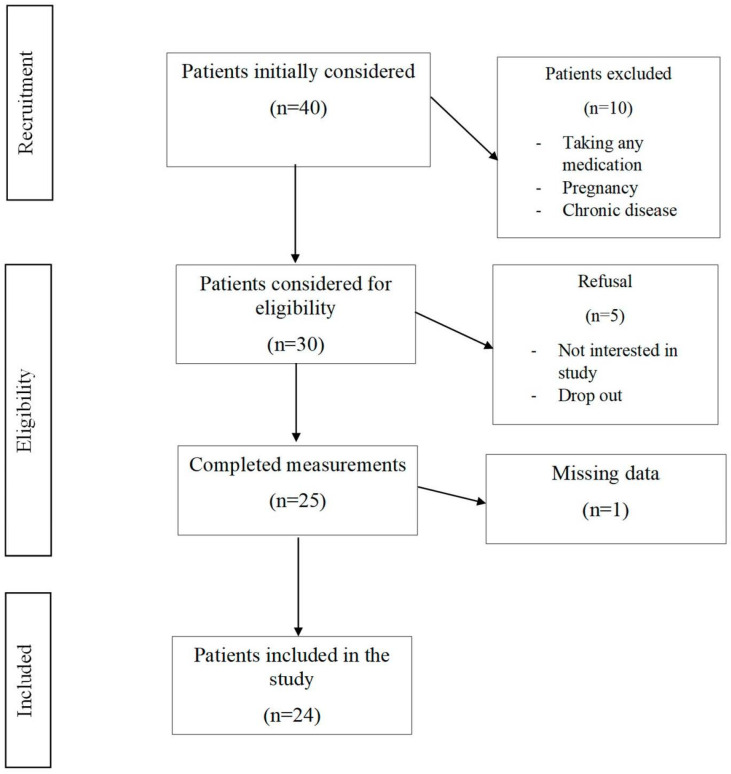
Flowchart of the included patients.

The study protocol was approved by the local Medical Ethical Committee (Prot.141/CE De Bellis 27 April 2021) and carried out in accordance with the Declaration of Helsinki (1964). Each participant provided written consent prior to enrolment. Patients were recruited from May 2021 to November 2022, and follow-up visits were scheduled during three clinical duties: at baseline (T0), after one and a half months, and after three months (T1). At T0 and T1, information was gathered, with fasting blood samples, anthropometric measurements, and instrumental performance (BIA and FibroScan). Additionally, patients were asked to complete a three-day food diary at both T0 and T1 to determine the number of daily servings of vegetables and carbohydrates. A summary flow diagram of the population screening process is shown in Figure 1.

Each patient was instructed to fill out a food diary for seven consecutive days. After evaluation of their usual food habits, the patients under study were invited to replace a common daily carbohydrate portion (bread, pasta, or potatoes) with a portion of 200 g of vegetables. Subjects were informed not to change their eating habits and lifestyle, except for the recommended change. The vegetables were four varieties of Brassicaceae supplied by the local agricultural enterprise “Spirito Contadino” free of charge for the patient. The four varieties of Brassicaceae are: Brassica rapa var. cymosa, Cichorium intybus, Brassica oleracea L. var. sabellica, and Sinapis arvensis var. orientalis. These vegetables are well-known sources of glucobrassicin-derivative molecules, such as isothiocyanates and phenolic compounds, which have shown antioxidant and antilipogenic effects in preclinical NAFLD data [20].

### 2.2. NAFLD Assessment

The FibroScan controlled attenuation parameter (CAP) was used to calculate the amount of ultrasound attenuation brought on by hepatic fat at the standard frequency of 3.5 MHz (Echosens, Paris, France). The cut-off chosen to distinguish the medium from the severe stage of steatosis was 300 dB/m [21]. Data analyses in obese patients suggest that CAP estimated with FibroScan is comparable to liver biopsy for the assessment and quantification of fatty liver. 

The FibroScan-AST (FAST) score is described as a way to identify people with an elevated probability of developing non-alcoholic fatty liver disease and non-alcoholic steatohepatitis [22]. It is obtained as follows: FAST = e^−1.65^ + 1.07 × ln(LSM) + 2.66 × 10^−8^ × CAP3 – 63.3 × AST − 1/1 + e^−1.65^ + 1.07 × ln(LSM) + 2.66 × 10^−8^ × CAP3 – 63.3 × AST − 1.

The FLI, a modelling approach that takes into account BMI, WC, triglycerides, and GT, was used to determine the likelihood of developing NAFLD [23]. The following algorithm was used: [e^0.953^ loge (TG) + 0.139 loge (BMI), 0.718 loge (GT), and 0.053 loge (WC)] 0.745/[1 + e^0.139^ BMI + 0.718 GT + 0.053 WC + 0.953 TG loge] 0.745 × 100.

### 2.3. Anthropometric Parameters

Height and weight were measured in fasting subjects wearing light clothing, without shoes, and with an empty bladder in order to calculate body mass index (BMI) (kg/m^2^), using the same stadiometer and scale for all. WC was measured in patients with an uncovered abdomen in an upright position at the midpoint between the lower edge of the ribs and the iliac crest. The neck measurement was also recorded. Extemporaneous diastolic (DBP) and systolic (SBP) blood pressures were measured three times each, with the patient sitting down, using an OMRON M6 automated blood pressure monitor.

### 2.4. Bioelectrical Impedance Analysis (BIA)

Bioelectrical impedance analysis (BIA) was carried out using a single-frequency bioimpedance analyzer (BIA-101 analyzer, 50-kHz frequency; Akern Bioresearch, Florence, Italy). According to the guidelines of the European Society of Parenteral and Enteral Nutrition, all participants were examined while lying down with their legs apart (ESPEN) [24]. For the 12 h before the test, they did not eat, exercise, or consume any alcohol. After removing shoes and socks, the contact areas were cleaned with alcohol prior to applying the electrodes. Injector electrodes were positioned at the metacarpal phalangeal joint on the dorsal surface of the right hand and at the transverse arch on the superior surface of the right foot. The sensor electrodes were placed on the distal prominence of the radius and ulna on the right wrist and the midpoint between the medial and lateral malleoli of the right ankle [25]. All measurements were made by an expert nutritionist in accordance with strict standards (SDN, MDC, RR). After obtaining Resistance (RZ) and Reactance (Xc), the software applied predictive equations to obtain FM, FFM, TBW, and ECW.

### 2.5. Biochemistry

After overnight fasting, blood samples were collected between the hours of 8:00 and 9:00 a.m. Serum was examined for fasting plasma glucose (FPG), haemoglobin A1c (HbA1c), insulin, lipid profile (total cholesterol, HDL cholesterol, and triglycerides), uric acid, and liver markers. The radioimmunoassay method (Behring, Scoppito, Italy) was used to measure the serum insulin concentrations using duplicate samples. To measure the serum concentrations of TSH, FT3, and FT4, a competitive luminometric technique based on the solid-phase antigen luminescent technology (SPALT) principle was applied (LIAISON FT3, FT4, TSH, DiaSorin, Saluggia, Italy). Fasting plasma glucose concentrations were determined using the glucose oxidase method, and fasting plasma lipid concentrations (triglycerides, total cholesterol, and HDL cholesterol) were measured using an automated colorimetric method (Sclavus, Siena, Italy) (Hitachi; Boehringer Mannheim, Mannheim, Germany). Glycated haemoglobin (HbA1c) was measured using an Architect c8000 chemical analyzer (Abbott). The URICASE/POD method was employed using an autoanalyzer to measure the amount of uric acid in the blood (Boehringer Mannheim). An automated system measured amino transferase, -glutamyl transpeptidase (GT), and creatinine in accordance with standard laboratory procedures (UniCel Integrated Workstations DxC 660i, Beckman Coulter, Fullerton, CA, USA). LDL cholesterol was calculated using the Friedewald equation [26]. DxI/Access was used to perform a quantitative analysis of serum ferritin (Beckman-Coulter AB, Bromma, Sweden). Chemiluminescence was used to measure the levels of serum 25(OH) vitamin D, and radioimmunoassay (Behring, Scoppito, Italy) to measure the levels of serum insulin (Diasorin Inc., Stillwater, OK, USA). The Homeostasis Model Assessment-Insulin Resistance (HOMA-IR) method was used to determine insulin resistance: [(fasting insulin fasting glucose)/405, normal range 0.23–2.5] [27]. Electrochemiluminescence analysis (ECLIA) was made to measure NT pro-BNP.

### 2.6. Data Management and Statistical Methods

Patients characteristics are reported as median and interquartile range (IQR) for continuous variables and as frequency and percentages (%) for categorical variables. The test of equality for matched data was used to compare differences between pairs of observation in the groups over time (before and after the vegetable diet) for continuous variables, while McNemar’s test was used for categorical variables. To test the null hypothesis of non-association, the two-tailed probability level was set at 0.05. Analyses were conducted with StataCorp. 2021 software. Release 17. College Station, TX: StataCorp LLC.

## 3. Results

Analysis of the epidemiological data of the patients, gathered using anamnestic questionnaires, revealed that the population examined (*n* = 24) was prevalently female (70.8%), and the age ranged from 21 to 62 years, with a median age of 47.5 (41.5–52.5) years; 42% (*n* = 10) of the enrolled patients had severe steatosis (CAP > 300 dB/m), while the remaining 58% (*n* = 14) had moderate steatosis (CAP < 300 dB/m). Only 4 patients smoked (17%). Concerning physical activity, investigated through the IPAQ, only 8% of the patients were active or very active (*n* = 2), so most people affected by steatosis were classified as sedentary (Table 1).

Anthropometric, metabolic, and hepatic parameters were compared before and after the nutritional intervention. The most significant finding from the analysis of liver parameters after intervention was an improvement of steatosis indices: the FLI index (*p* < 0.0001) and the FAST score (*p* = 0.007) derived from elastography both decreased significantly compared to T0 (Table 2).

Following dietary changes for three months, the basic anthropometric indicators diminished as expected, i.e., BMI, waist circumference (WC), neck circumference, and fat mass (FM) were all significantly lower (*p* < 0.0001), as well as extracellular water (ECW) (*p* = 0.03). On the other hand, there were no significant changes in fat free mass (FFM) and skeletal muscle mass (SSM) (*p* > 0.05) (Table 2). As regards metabolic parameters, there was a significant reduction in HbA1c (*p* = 0.01), triglycerides (*p* = 0.03), as a result of restricting simple sugars and consuming more fibre, which decreases the absorption of sugars and fats. Although not statistically significantly, the values for HOMA-IR and insulin decreased in accordance with the drop in glycated haemoglobin (*p* = 0.06). Liver markers including AST, ALT, and GGT all decreased, in line with FLI and FAST findings, but only the first two significantly (*p* = 0.01; *p* = 0.02; *p* = 0.06). Additionally, a decrease in NT-proBNP was found (*p* = 0.03), probably due to the increased intake of magnesium and potassium from vegetables. Lastly, a reduction in TSH (*p* = 0.02) and an increase in albumin (*p* = 0.0005) were observed, possibly due to an improved general nutritional status (Table 2). A correlation matrix was carried out to show that the metabolic improvements obtained are actually attributable to diet quality and not just weight loss (Table S1).

## 4. Discussion

The healthy benefits following a lifestyle modification in patients with NAFLD are widely recognized, but the major limitation to achieve a reliable and sustained goal is maintaining adherence to the diet, which is inversely related to the impact of the restrictions. Herein, we demonstrated for the first time that just one single daily diet change is enough to improve, after only three months, the degree of fibrosis in patients with NAFLD. In particular, the replacement of a daily carbohydrate portion with 200 gr of brassicaceous vegetables improves the circulating biomarkers linked to NAFLD, the FLI, and the FibroScan score. These results are explained by sugar restriction, which both downregulate the de novo lipogenesis (DNL) widely recognized in NAFLD [28,29] and reduce inflammatory responses [30]. Furthermore, our findings support recent research showing an inverse relationship between leafy green vegetable consumption and NAFLD [31]. We observed that the FAST-score, a FibroScan-derived indirect predictor of steatohepatitis, was significantly lower after the assigned diet change than at time zero. However, it must be noted in consideration that while liver fat indices have been effective in the early diagnosis of NAFLD and may be used to assess the status of the liver cross-sectionally, their ability to accurately represent interventional changes in liver fat content appears to be diet specific [32,33]. However, consistently, in line with other previous research, increasing vegetable intake was linearly associated with a significant drop in the FLI index [34]. Green leafy vegetables are rich in dietary fibers, which have several healthy effects on NAFLD weight loss. Firstly, they increase satiety, thereby encouraging calorie restriction, and, secondly, they slow the absorption of sugars and fats [35]. Moreover, after being fermented by the gut microbiota, soluble fibre results in the production of short-chain fatty acids, which feed and modify the gut bacteria. This prebiotic property contributes to the treatment of NAFLD, based on studies showing that dysbiosis of the gut microbiota and small bowel bacterial overgrowth may be associated with the onset of NAFLD [13]. 

The presence of several bioactive substances, including ascorbic acid, phenolics, carotenoids, and glucosinolates may be responsible for plant health benefits, such as modulating the inflammatory response, reducing ROS (with scavenger activity), and decreasing lipid oxidation [14]. Recently, different studies reported favourable effects of polyphenols derived from Brassicaceae on the gut-liver-adipose axis, a known regulator of NAFLD [20]. Furthermore, there is an inverse correlation between carotenoid intake and the onset of NAFLD. The exposure of hepatocytes to higher concentrations of fatty acids during NAFLD pathogenesis may lead to the production of reactive oxygen species that induce lipid peroxidation and cytokine release, damaging hepatocytes. Carotenoids have well-known anti-oxidant properties, which include suppressing free radicals, minimising injuries caused by reactive oxidant species, and inhibiting lipid peroxidation [36]. Magnesium, which is abundant in vegetables, is known to increase insulin sensitivity, a crucial factor in lowering the risk of NAFLD [37]. Consumption of magnesium was also inversely correlated with specific metabolic syndrome markers, including fasting glucose levels and waist circumference [38]. This is consistent with our results and with those described by Chen et al., showing a reduction of weight, fat mass, and waist circumference in patients with NAFLD following two months on a low-carb, high-fibre diet [39]. 

The consumption of cruciferous vegetables, as well as the reduced intake of carbohydrates, are both linked to a lower risk of type 2 diabetes [40]. Moreover, we found that triglyceride levels were decreased following the dietary intervention, most likely as a result of the presence of vegetable fibres, which are known to lessen the absorption of fats and simple sugars [41]. An interesting result was that NT-proBNP, a marker of cardiac strain, decreased as vegetable consumption increased, and this effect may be explained by the higher contents of fibres, potassium, and magnesium in vegetables [42]. Lastly, NT-proBNP levels were lower after the dietary intervention, and this is an interesting result when considering that NT-proBNP levels are predictive of an adverse long-term outcome in patients with and without known heart failure [43]. TSH was found to be lower after weight loss. This might be a result of both the altered pituitary energy expenditure and the altered levels of leptin and adipose tissue [44,45]. 

### Strengths and Limitations

The main strengths of this study are the homogenous characteristics of the study population, and the exclusion of subjects taking any medication, thus avoiding possible confounder effects; the longitudinal prospective design of the study; the objective measurements of liver fibrosis; and the novelty in the literature of the specific impact of even a small amount of vegetables on NAFLD. Furthermore, the study demonstrates that the metabolic improvement achieved is actually driven by the quality of the diet and not by weight loss. The limitations of the study include the sample size, which allowed us to extrapolate only preliminary correlations, and the single-centre nature of the study.

## 5. Conclusions

We conclude that a simple and easily acceptable dietary change such as swapping a standard serving of vegetables to replace one portion of carbohydrates improves NAFLD and metabolic markers such as glycated haemoglobin and triglycerides, and facilitates weight loss. 

## Figures and Tables

**Table 1:** Baseline characteristics of patients following the nutritional intervention (*n* = 24).

Parameters *	Median (IQR) or Frequency (%)
Gender (F) (%)	17 (70.8)
Age (years)	47.5 (41.5–52.5)
Smoker (Yes) (%)	4 (16.7)
IPAQ	1032 (480–1701)
IPAQ (%)	
Inactive	7 (29.2)
Sufficiently Active	15 (62.5)
Active or Very Active	2 (8.3)
Snoring (Yes) (%)	17 (70.8)
PREDIMED score	8 (7–8)

* As Median and Interquartile Range (IQR) for continuous variables, and as frequency and percentage (%) for categorical variables. Abbreviations: IPAQ, International Physical Activity Questionnaire; PREDIMED, PREvención con DIeta MEDiterránea.

**Table 2:** Effect of the nutritional intervention on anthropometric, blood, and liver parameters.

Parameters *	Vegetable Diet		*p* ^^^
	Before	After	
*Antropometric*			
Weight (kg)	95.0 (83.0–109.2)	89.7 (75.2–103.0)	**<0.0001**
BMI (kg/m^2^)	35.3 (31.2–39.0)	33.3 (28.6–37.3)	**<0.0001**
Waist (cm)	110.0 (103.0–124.0)	106.5 (95.0–112.5)	**<0.0001**
Neck (cm)	39.5 (38.0–42.5)	38.0 (35.0–41.5)	**<0.0001**
Systolic Blood Pressure (mmHg)	130 (130–137)	127 (120–135)	0.11
Diastolic Blood Pressure (mmHg)	85 (80–90)	82 (75–90)	0.24
*Blood*			
FPG (mg/dL)	94 (89–98)	90 (86–99)	0.99
Insulin (µUI/mL)	14.0 (10.4–19.8)	10.5 (8.6–14.7)	0.06
HOMA-IR (mg/dL)	3.0 (2.5–4.3)	2.5 (1.9–3.5)	0.06
HbA1c (mmol/mol)	38.0 (34.0–40.5)	36.0 (33.5–39.0)	**0.01**
Triglycerides (mg/dL)	90 (64–132)	72 (62–90)	**0.03**
LDL (mg/dL)	112 (97–135)	110 (96–123)	0.99
HDL (mg/dL)	51 (43–58)	54 (49–61)	0.40
Total Cholesterol (mg/dL)	193 (161–216)	181 (158–197)	0.29
TSH (µUI/mL)	1.8 (1.4–2.3)	1.5 (1.2–2.0)	**0.02**
FT3 (pg/mL)	3.3 (3.0–3.6)	3.3 (2.8–3.5)	0.84
FT4 (ng/mL)	1.1 (1.0–1.3)	1.2 (1.1–1.2)	0.31
Vitamin D (ng/mL)	22.1 (19.5–26.2)	23.8 (16.9–28.9)	0.54
Uric acid (mg/dL)	4.9 (4.1–6.2)	5.0 (4.1–5.9)	0.82
Creatinine (mg/dL)	0.7 (0.7–0.9)	0.8 (0.7–0.9)	0.38
GFR (mL/min/1.73 m^2^)	89 (73–90)	86 (73–90)	0.18
AST (U/L)	18 (15–27)	17 (14–19)	**0.01**
ALT (U/L)	19 (13–30)	17 (12–22)	0.06
GGT (U/L)	16 (14–27)	16 (13–20)	**0.02**
Ferritin (ng/mL)	52.7 (29.3–151.5)	63.5 (40.9–139.0)	0.54
PTH (pg/mL)	56.3 (46.8–56.3)	53.0 (44.8–53.0)	0.06
NT proBNP (pg/mL)	47 (34–52)	46 (34–46)	**0.03**
Albumin (g/dL)	4.2 (4.2–4.2)	4.3 (4.3–4.4)	**0.0005**
*Liver*			
Fibroscan CAP (dB/m)	278 (253–354)	278 (200–331)	0.84
Fibroscan CAP (%)			0.69 ^¥^
≤300 dB/m	14 (58.33)	16 (66.67)	
>300 dB/m	10 (41.67)	8 (33.33)	
Fibroscan E (kPA)	5.3 (4.1–6.7)	4.9 (4.3–5.9)	0.68
FM (kg)	37.9 (27.7–43.5)	32.3 (23.4–40.7)	<0.0001
FFM (kg)	54.3 (50.0–68.4)	53.0 (48.5–66.3)	0.31
TBW (L)	40.0 (37.3–50.5)	39.1 (35.4–48.7)	0.15
ECW (L)	18.3 (15.9–22.7)	17.3 (15.2–20.8)	**0.03**
SMM (kg)	29.7 (23.4–36.7)	30.3 (24.0–35.6)	0.99
FLI	85 (54–95)	73 (33–89)	**<0.0001**
FAST	0.05 (0.02–0.15)	0.03 (0.02–0.09)	**0.007**

* As Median and Interquartile Range (IQR) for continuous variables, and as frequency and percentage (%) for categorical variables. ^^^ Wilcoxon matched-pairs signed-rank test; ^¥^ McNemar-Bowker test. Abbreviations: BMI, Body Mass Index; HOMA-IR, Homeostasis Model Assessment-Estimated Insulin Resistance; HbA1c, Glycosylated Hemoglobin; HDL, High-Density Lipoprotein; TSH, Thyroid-stimulating hormone; FT3, Free Triiodothyronine; FT4, Free Thyroxine; GFR, Glomerular Filtration Rate; AST, Aspartate Transaminase; ALT, Alanine Amino Transferase; GGT, Gamma Glutamyl Transpeptidase; PTH, Parathyroid Hormone; NT proBNP, N Terminal Pro-B-type Natriuretic Peptide; Fibroscan CAP, Fibroscan Controlled Attenuation Parameter; Fibroscan E, Fibroscan Transient Elastography; FM, Fat Mass; FFM, Lean Mass; TBW, Total Body Water; ECW, Extracellular Hydration; SMM, Skeletal Muscle Mass; FLI, Fatty Liver Index; FAST, FibroScan-AST.

## Data Availability

The original contributions presented in the study are included in the article. Further inquiries can be directed to the corresponding author.

## Supplementary Materials

The following supporting information can be downloaded at: https://www.mdpi.com/article/10.3390/nu15102289/s1, Table S1: Correlation ^¥^ between weight loss and blood and liver parameters.
