# Supplementary material for: The Replacement of Only One Portion of Starchy Carbohydrates with Green Leafy Vegetables Regresses Mid and Advanced Stages of NAFLD: Results from a Prospective Pilot Study
Source: Nutrients. 2023 May 12;15(10):2289. doi: 10.3390/nu15102289 (PMC10222437; doi:10.3390/nu15102289)
Supplement: Supplementary file 1 [file nutrients-15-02289-s001.zip › nutrients-2343157-supplementary.pdf]

Table S1. Correlation [¥] between weight loss and blood and liver parameters.

Parameters [*]	
FPG (mg/dL)	0.15 (0.47)
Insulin (µUI/mL)	0.16 (0.46)
HOMA-IR (mg/dL)	0.15 (0.48)
HbA1c (mmol/mol)	0.06 (0.78)
Triglycerides (mg/dL)	0.24 (0.26)
LDL (mg/dl)	-0.28 (0.19)
HDL (mg/dl)	-0.24 (0.27)
Total Cholesterol (mg/dl)	-0.07 (0.75)
TSH (µUI/mL)	0.02 (0.93)
FT3 (pg/mL)	0.26 (0.22)
FT4 (ng/mL)	0.18 (0.40)
Vitamin D (ng/mL)	0.13 (0.54)
Uric Acid (mg/dL)	0.02 (0.91)
Creatinine (mg/dL)	-0.15 (0.48)
GFR (mL/min/1.73 m^2)	0.10 (0.63)
AST (U/L)	0.16 (0.46)
ALT (U/L)	0.27 (0.20)
GGT (U/L)	0.31 (0.14)
Ferritin (ng/ml)	-0.14 (0.51)
PTH (pg/mL)	0.17 (0.42)
proBNP (pg/mL)	-0.09 (0.66)
Albumin (g/dL)	-0.03 (0.88)
Fibroscan CAP (dB/m)	0.25 (0.24)
Fibroscan E (kPA)	-0.09 (0.67)
**FM (Kg)**	**0.63 (0.001)**
FFM (Kg)	0.04 (0.84)
TBW (L)	0.10 (0.62)
ECW (L)	0.02 (0.94)
SMM (Kg)	-0.03 (0.88)
**FLI**	**0.68 (0.0004)**
FAST	0.06 (0.78)

[¥] rho, Spearman correlation coefficient.
